# Supplementary material for: Salt Stress-Induced Modulation of Porphyrin Biosynthesis, Photoprotection, and Antioxidant Properties in Rice Plants (Oryza sativa)
Source: Antioxidants (Basel). 2023 Aug 15;12(8):1618. doi: 10.3390/antiox12081618 (PMC10451626; doi:10.3390/antiox12081618)
Supplement: Supplementary file 1 [file antioxidants-12-01618-s001.zip › antioxidants-2551695-supplementary.pdf]

Table S1. Primers used for RT-qPCR assays.

Gene	Primer sequence
NHX1	F: TGGCTGCTGCTAATGAGTTG
	R: CAGGGTGGCAACTAGGAAAG
SOS1	F: CTACCACCAAATGCCGACTT
	R: ATTCTGAGCTCCCCTGGATT
LHCB1	F: CAGCTCTCACAGCTCACTGC
	R: GTGTCCCACCCGTAGTCG
LHCB6	F: CTCATGGGCTGGGTAGAGTC
	R: CGGCCTCGAAGTAGAAGATG
RBCS	F: GCAGCTTTGTTTTGGGCTAT
	R: CCGGAGCTTTGTTTTCACAT
BCH	F: CAACCGGAGCTTGTGATTTT
	R: TACAATGTTCAGCCGCAGAG
VDE	F: GAAATGCGTCCCACAAAAGT
	R: TTATAGAGGATCGCGGGTTG
HEMA1	F: GCTATGGGTGGTGTTCGACT
	R: CGATCTTCTGGAGGCACTTC
GSA	F: CTCCGTGACTTGACGAAACA
	R: GTAGGTTCCAGGCTCCATCA
ALAD	F: GTCCACCGTCTCCTTCTCC
	R: TGTCAAGTCAAGAGGCCTGA
PPO1	F: ACAGTTCCTCATTGGCCATC
	R: CCCATGAAATTTTTGCTGCT
CHLD	F: TGGGACAGCAAAGACAGTGA
	R: AAGGCCAGGTTGAAACACAG
CHLH	F: GTGTGGGTTGCGTTCTTTTT
	R: GGTGACAATGTGGCTCCTCT

CHLI	F: TGTGCTTCTGGATTCTGCTG
	R: GCTGGAGCTTGTCTTGTTCC
PORB	F: GTGAATTGCCAGGTTTTCGT
	R: GCAATTAGCAAAGCTGCACA
FC2	F: TTGGTGCTATGGCAGTTTCA
	R: AGTGGAACAAAGGCAGGATG
HO1	F: AGCGCTAGCAGTAGCAGGAG
	R: GCTCCTTCTCCCCTTCCTT
HO2	F: AGGGACCTAGCAGCCCTAAC
	R: CCCGTATCGTCCATCTTGAG
APXa	F: ACAAAGCCCTGCTGAGTGAC
	R: TAACAGCCCACCGAGACATT
APXb	F: CCAAGTGACAAAGCCCTCAT
	R: TCTTGACAGCAAATAGCTTGG
CATa	F: CCAGTGTGATGAGTCGTTGG
	R: ATATGCAGGCTCCATTTTGG
CATb	F: AAGGATGGAAAGCCTCACCT
	R: AGGGGGATGATATCCTCTGG
CATc	F: ACAACCACTACGACGGCTTC
	R: CCAGTAGGAGAGCCAGATGC
Actin	F: CTTCATAGGAATGGAAGCTGCGGGTA
	R: CGACCACCTTGATCTTCATGCTGCTA

F, forward; R, reverse.
